# Relationship between Medicaid coverage design and receipt of medication for alcohol use disorder (MAUD): Probability of receipt increases based on comprehensiveness of plan

**DOI:** 10.1016/j.dadr.2025.100374

**Published:** 2025-08-22

**Authors:** Miguel Antonio Garcia Estrada, Shelby R. Steuart, Christina M. Andrews, Colleen M. Grogan, Olivia M. Hinds, Emily C. Lawler, Felipe Lozano-Rojas, Melissa A. Westlake, Lauren Peterson, Coady Wing, Amanda J. Abraham

**Affiliations:** a Department of Public Administration and Policy, University of Georgia, United States; b Crown Family School of Social Work, Policy, and Practice, The University of Chicago, United States; c Department of Health Services Policy and Management, Arnold School of Public Health, University of South Carolina, United States; d Center for Health Administration Studies, The University of Chicago, United States; e National Bureau of Economic Research, United States; f School of Public and Environmental Affairs, Indiana University, United States

**Keywords:** Alcohol use disorder, Medicaid managed care, Medications for alcohol use disorder (MAUD)

## Abstract

Alcohol use disorder (AUD) affects one in ten Americans. As one of the largest payers of AUD treatment in the United States, Medicaid managed care plays a key role in facilitating access to AUD treatment services and medications. However, little is known about how AUD coverage in Medicaid managed care organizations (MCOs) affects treatment receipt. We examined the relationship between the comprehensiveness of Medicaid MCO plan coverage of AUD treatment and receipt of medications for AUD (MAUD). We used Medicaid claims data from Kentucky (2016–2019); our final analytic sample consisted of 202,230 newly enrolled Medicaid beneficiaries. Kentucky quasi-randomly assigns Medicaid beneficiaries to one of five MCO plans with different AUD treatment coverage. We leveraged the random assignment to MCO plans using a Two-Stage Least Squares/Instrumental Variable (TSLS/IV) approach to estimate the effects of MCO plan comprehensiveness on receipt of MAUD. Diagnosis with AUD and receipt of MAUD was relatively uncommon— only 0.5 % of Medicaid beneficiaries were diagnosed with AUD and received MAUD across all plans. Results showed that for each additional AUD treatment modality covered, the probability of receiving MAUD increased by 6.7 % relative to the mean [mean: 0.5 %; difference per additional service/MAUD (in percentage points): 0.033; p < 0.05]. Expanding coverage in the least comprehensive MCO plan to match the most comprehensive plan would increase the probability of receiving MAUD by 47 %. Overall, study findings indicate that when insurance plans cover a broader array of AUD treatment services and medications, patients are more likely to receive MAUD.

## Highlights

• Coverage of AUD treatment varies across Medicaid managed care organization plans.
• We examine whether comprehensiveness of AUD treatment coverage affects MAUD receipt.
• Comprehensive coverage of AUD treatment increased the probability of receiving MAUD.
• Improving comprehensiveness of AUD treatment coverage may accelerate MAUD uptake.

## 1 Introduction

Excessive alcohol consumption is among the leading causes of preventable disease and death in the United States, with an estimated 178,000 related deaths in 2021 (Esser et al., 2024). Alcohol use disorder (AUD), a chronic health condition affecting roughly one in ten individuals, results in numerous chronic and acute health concerns and increases risk of premature death (Center for Behavioral Health Statistics and Quality, 2024). Deaths from excessive alcohol use in the United States increased from 38.1 deaths per 100,000 population in 2016–2017–47.6 deaths per 100,000 population in 2020–2021, representing an increase of 29.3 % (Esser et al., 2024). Although effective treatment for AUD treatment exists, low rates of treatment for the condition persist due to a wide range of barriers to treatment access, including stigma, low screening rates in primary care, and health care costs (Carvalho et al., 2019, Kranzler and Soyka, 2018, Mekonen et al., 2021). Indeed, financial constraints, including limitations in health insurance coverage for AUD-related services, have long been cited as an important factor that can hinder access to AUD treatment (Priester et al., 2016, Schuler et al., 2015).

Medicaid provides health insurance to roughly 70 million people (KFF, 2023) or one in five Americans, and is the largest single payer of AUD treatment in the United States (Centers for Medicare and Medicaid Services, 2022, Grant et al., 2017; KFF, 2021; Olfson et al., 2018). Within Medicaid, managed care organizations (MCOs) contract with states to administer benefits for roughly 75 % of all Medicaid enrollees nationwide (KFF, 2022). As a result, Medicaid MCOs play an outsized role in shaping the delivery of AUD care. Medicaid MCOs have substantial discretion over benefit design for AUD treatment, including the specific treatment services and medications MCO plans elect to cover.

Existing research shows that Medicaid MCO plan features vary considerably (Medicaid and CHIP Payment and Access Commission, 2019, Medicaid and CHIP Payment and Access Commission, 2023; Peterson et al., 2024) and that coverage of AUD treatment services and medications differs across individuals enrolled in different MCO plans in the same state (Stewart et al., 2025b). It is likely that differences in AUD treatment coverage across MCO plans affects utilization of AUD treatment, including MAUD. MCO plans that cover a wider range of services (e.g., detoxification, outpatient, intensive outpatient, residential treatment) offer individuals multiple points of entry into AUD treatment, which may help facilitate receipt of MAUD. For example, an MCO plan that offers coverage across the continuum of care (e.g., detoxification, outpatient, intensive outpatient, residential treatment) provides a broader array of treatment modalities through which individuals can engage with the treatment system and initiate MAUD, compared to an MCO plan that covers only outpatient treatment, which engages patients with less frequency and may be not be appropriate for individuals requiring more intensive support. Likewise, more comprehensive coverage of MAUD is critical to deliver patient-centered care for AUD because each MAUD differs in terms of clinical outcomes/targets, dosing regimen, and cost (Reus et al., 2018).

To date, no prior studies have examined the relationship between Medicaid MCO AUD treatment coverage and MAUD receipt. One previous study documented variation in AUD coverage across Medicaid MCO plans but the study did not consider the effects of coverage on receipt of AUD treatment (Stewart et al., 2025b). A small literature has begun to examine the effects of Medicaid MCO coverage of substance use disorder (SUD) treatment, focusing primarily on the use of medications for opioid use disorder (Abraham et al., 2022, Stewart et al., 2025a). However, existing studies of the association between Medicaid MCO coverage and SUD treatment use rely on research designs that do not account for the possibility that Medicaid beneficiaries may select into MCO plans partly on the basis of their individual AUD/SUD risk or their individual demand for AUD/SUD treatment. This type of self-selection could lead to biased estimates of the true effects of MCO plan coverage features on treatment utilization.

To address these gaps in the literature, we examine the impact of MCO plan comprehensiveness of AUD treatment coverage on receipt of MAUD in Kentucky – a state Medicaid program administered entirely by managed care. A key feature of our study is that Kentucky quasi-randomly assigns newly enrolled beneficiaries to one of five MCO plans. Because of random assignment, plan characteristics are not correlated with individual level AUD risk, treatment demand, or any other patient characteristics at baseline. Leveraging this quasi-random assignment to MCO plans, we address the following research question: Does enrollment in a Medicaid MCO plan with more comprehensive coverage for AUD treatment services and medications increase the probability of a Medicaid beneficiary receiving MAUD?

## 2 Methods

### 2.1 Data and study population

We used data on Kentucky Medicaid beneficiaries from the 2016–2019 T-MSIS Analytic Files (TAF), which include all paid and adjudicated Medicaid claims in the state. Beginning in 2011, Kentucky Medicaid started operating entirely under a managed care system. Upon enrollment, beneficiaries are automatically assigned to one of five MCO plans: Aetna Better Health of Kentucky, Anthem, Humana CareSource, Passport Health Plan, and WellCare of Kentucky. Plan assignment is determined by an algorithm that is random, conditional on several factors: household members’ plan assignment, load balancing across plans, and prior physician relationships (Palmer et al., 2012). Although initial assignment is random and automatic, beneficiaries may opt to switch from their assigned plan to another plan, provided they do so within a 90-day period following enrollment.

We used the TAF Medicaid Managed Care Supplement file to identify beneficiaries’ auto-assigned plan and to determine whether they switched to another plan within 90 days of enrollment. In each year from 2017 to 2019, we limited our sample to people aged 18–64 who were newly enrolled in Medicaid in a given year and who were not enrolled in Kentucky Medicaid in the previous year.

We focused on two analytic study populations. The *one-year sample* consisted of all newly enrolled individuals in 2017, 2018, and 2019 who had at least three months of continuous coverage and did not have breaks in coverage for more than one month (n = 202,230). The *two-year sample* consisted of the subset of newly enrolled individuals in 2017 or 2018 who remained continuously enrolled for two years with at least three months of continuous coverage and no breaks in coverage for more than one month (n = 119,486). The average number of days of enrollment per calendar year was largely consistent across MCO plans, with an average of 189.4 days of enrollment per calendar year (range: 186.3–195.2 days). For sample sizes across study years, see Appendix Table 1.

#### 2.1.1 Dependent variable

Our primary dependent variable was a dichotomous variable coded as ‘1’ if a beneficiary was *both* diagnosed with AUD *and* received MAUD within one year of enrollment (or within two years of enrollment for the two-year sample). We used ICD-10 codes from the Centers for Medicare and Medicaid Services (CMS) Chronic Conditions Warehouse (CCW) algorithm to identify individuals with an AUD diagnosis (Chronic Conditions Warehouse, 2024, Hu et al., 2022). Beneficiaries who received MAUD were identified if they had a claim that included an AUD diagnosis concurrent with national drug codes for acamprosate, disulfiram, injectable naltrexone, and oral naltrexone, or an MAUD-related HCPCS code from CMS (Centers for Medicare & Medicaid Services, n.d.). This approach of identifying MAUD receipt based on whether an individual had both an AUD diagnosis and any MAUD helps ensure that medications were intended for AUD and not for other health conditions. For example, naltrexone, one of the FDA-approved medications for AUD, is also used in the treatment of opioid use disorder.

#### 2.1.2 Key independent variable

Our key independent variable measured the *comprehensiveness* of AUD treatment coverage in a beneficiary’s assigned MCO plan. To construct the comprehensiveness score, we first measured the total number of AUD treatment services and medications a plan covered in each year. Then, we centered each MCO plan’s AUD treatment services and medications coverage count around the coverage count of the MCO plan with the least comprehensive coverage. Thus, the index was set to 0 for the least generous MCO plan and increased incrementally for each additional service or medication covered.

Data on plan-specific coverage was sourced and coded following a thorough review of MCO plan documents (member handbook, provider manual, prescription drug formularies) for all active Kentucky MCO plans (Abraham et al., 2022, Andrews et al., 2024, Silverman et al., 2024). Treatment services included outpatient, intensive outpatient/partial hospitalization, outpatient or inpatient detoxification, inpatient, residential, and recovery support services. Medications included all FDA-approved medications for AUD treatment— acamprosate, disulfiram, oral naltrexone, and injectable naltrexone (Substance Abuse and Mental Health Services Administration, 2015). The treatment services and medications selected for inclusion in this study were guided by the continuum of care for SUD treatment recommended by the American Society of Addiction Medicine (ASAM) (Mee-Lee, 2013).

#### 2.1.3 Covariates

Our main regression models adjusted for county and year fixed effects as well as a set of baseline covariates: sex (female=1, male=0), household size, race/ethnicity (Hispanic, Black, White [referent], missing race/ethnicity), and age category (18–33, 34–49, 50–64 [referent]). Controlling for race and ethnicity was based on documented differences in the literature in the likelihood of receiving MAUD across different racial and ethnic groups. For example, two prior studies found that non-Hispanic White patients were more likely to receive MAUD, compared to Hispanic patients (Han et al., 2021, Hu et al., 2022) and another more recent study found that Black patients were less likely to receive an MAUD order, compared to White patients (Hodgkin et al., 2024).

### 2.2 Statistical analysis

Random assignment implies that both measured and unmeasured baseline covariates should be balanced across initial MCO plan assignments, and this assumption is crucial to the validity of our research design. To assess balance, we calculated Cohen’s D statistics for each baseline covariate for each assigned plan-year compared with the state average across all plans in that year, separately for beneficiaries living in the two large urban counties of Kentucky (Jefferson [Louisville] and Fayette [Lexington]) and beneficiaries living outside of these two counties (See Appendix Table 3–6). Cohen’s D statistics were calculated separately for the two largest urban counties versus the rest of the counties in the state because one plan (Passport) had a higher proportion of Black enrollees compared to the state average which was due to the plan’s higher enrollment of beneficiaries in the state’s two largest urban counties. Results showed that once stratified by geography the covariates were well balanced across plans: Cohen’s D was below 0.15 in absolute value for all but one comparison of 21 covariates, five plans, and four sub-populations. Thus, the balancing tables support our core assumption that initial MCO plan assignment was random and was not correlated with baseline characteristics, even after adjusting for county and year fixed effects. Moreover, we conducted a sensitivity analyses stratified by geography (two-largest urban counties versus the rest of the counties in the state) to examine the robustness of our main results, and the results were consistent with our main findings (See Appendix Table 7).

For each beneficiary, two measures of MCO plan comprehensiveness were used in the analyses: “assigned” plan comprehensiveness and “realized” plan comprehensiveness. Assigned plan comprehensiveness was defined as the comprehensiveness of the initial auto-assigned plan, while realized plan comprehensiveness was defined as the comprehensiveness of the final observed plan, following the 90-day period during which plan switching was allowed (i.e., the realized plan). Hence, comprehensiveness scores differed between assigned and realized plans only if the beneficiary switched plans following initial auto-assignment.

Assigned and realized MCO plan comprehensiveness were observed for our two study samples. For the one-year sample, we defined each beneficiary’s assigned and realized plans based on his/her first versus final observed MCO plan within the first year of enrollment. For the two-year sample, we followed beneficiaries continuously enrolled for two years and identified assigned plan comprehensiveness based on the first observed plan during the first year of enrollment, and realized plan comprehensiveness based on the final observed plan during the second year of enrollment. In our two study samples, between 86.6 % and 94.4 % of beneficiaries stayed in their auto-assigned plan (See Appendix Table 2).

Given that a small proportion of beneficiaries switched out of their auto-assignment plan (average of 5.0 % for the one-year sample; 9.3 % for the two-year sample), and this choice may be correlated with individual AUD risk or demand for AUD treatment, we used a Two-Stage Least Squares (TSLS)/Instrumental Variable (IV) approach. In the first stage, we regressed realized plan comprehensiveness (dependent variable) on assigned plan comprehensiveness (independent variable). In the second stage, to assess the impact of plan comprehensiveness, we regressed MAUD receipt (dependent variable) on predicted plan comprehensiveness (independent variable) from the first stage.

We conducted the regression analyses using several different specifications: (1) without covariates and fixed effects, (2) without covariates but with county and year fixed effects, and (3) with covariates and county and year fixed effects. Use of county and year fixed effects accounted for potential confounders related to time-invariant characteristics and common trends across counties, respectively. The use of baseline covariates accounted for observable differences across counties.

We also conducted analyses examining AUD diagnosis as the outcome variable (Appendix Table 8), analyses using an Intent-to-treat (ITT) estimator (Appendix Table 9), and sensitivity analyses in which the outcome was modeled using logistic regression and the instrumental variable was incorporated using a Two-Stage Residual Inclusion estimator (Appendix Table 12). Results using the Two-Stage Residual Inclusion estimator were similar to our main results, suggesting that our main analysis was not sensitive to assumptions about the non-linear form of the outcome variable. All analyses were conducted using Stata 18.0.

## 3 Results

The study population’s characteristics were similar for the one- and two-year samples. Approximately 51.4 % of beneficiaries in the one-year sample and 51.6 % in the two-year sample were female (Table 1). An estimated 61.8 % and 64.3 % in the one- and two-year samples were White, respectively, while 11.0 % and 10.9 % of beneficiaries were Black. Moreover, about 47.3 % of beneficiaries in the one-year sample were aged 18–33 and 47.8 % of beneficiaries were aged 18–33 in the two-year sample. The percentage of beneficiaries diagnosed with AUD and receiving MAUD was lower for beneficiaries in the one-year sample (0.2 %; n = 404) compared to those in the two-year sample (0.5 %; n = 597).

**Table 1:** Summary statistics.

**Variable**	**(1)** **One-year Sample**		**(2)** **Two-year Sample**	
	**%**	**n**	**%**	**n**
Female	51.4	103,946	51.6	61,655
Household Size (Mean, SD)	1.8	1.3	1.9	1.3
Race/Ethnicity				
White	61.8	124,978	64.3	76,829
Hispanic	3.5	7078	2.6	3107
Black	11.0	22,245	10.9	13,024
Missing Race/Ethnicity	22.0	44,491	20.5	24,495
Age Groups				
18–33	47.3	95,655	47.8	57,114
34–49	31.0	62,691	31.6	37,758
50–64	21.7	43,884	20.6	24,614
MAUD Receipt	0.2	404	0.5	597
Observations	202,230		119,486	

On average, MCO plans covered seven AUD treatment services and/or medications per year, with a range from three to 10 (mean: 6.7; SD: 1.9) (Table 2). Passport (mean: 9.7; SD: 0.5) covered the greatest number of AUD treatment services and medications, on average, while Aetna (mean: 4.7; SD: 1.2) covered the lowest number. With the exception of Aetna, all MCO plans covered at least three FDA-approved medications for treatment of AUD over the study period, and the number of medications covered by each plan was consistent across years. However, coverage of AUD treatment services varied substantially across plans and years. Hence, variation in plan comprehensiveness largely reflects differences in AUD treatment service coverage rather than MAUD coverage.

**Table 2:** MCO comprehensiveness of coverage for AUD treatment, by plan and year.

**Year**	**Aetna**	**Anthem**	**Humana**	**Passport**	**Wellcare**
2017	**Total: 3** MAUD (2) Services (1)	**Total: 6** MAUD (3) Services (3)	**Total: 8** MAUD (4) Services (4)	**Total: 9** MAUD (4) Services (5)	**Total: 5** MAUD (3) Services (2)
2018	**Total: 5** MAUD (2) Services (3)	**Total: 5** MAUD (3) Services (2)	**Total: 7** MAUD (4) Services (3)	**Total: 10** MAUD (4) Services (6)	**Total: 7** MAUD (3) Services (4)
2019	**Total: 6** MAUD (2) Services (4)	**Total: 5** MAUD (3) Services (2)	**Total: 7** MAUD (4) Services (3)	**Total: 10** MAUD (4) Services (6)	**Total: 7** MAUD (3) Services (4)
**Overall** **(within plan)**	**Mean: 4.7** **SD: 1.2**	**Mean: 5.3** **SD: 0.5**	**Mean: 7.3** **SD: 0.5**	**Mean: 9.7** **SD: 0.5**	**Mean: 6.3** **SD: 0.9**
**Overall (across plans)**	**Mean: 6.7, SD: 1.9**				

Note: Medications and services which were “not specified” in the MCO plans were coded as ‘0’ (not covered).

**Fig. 1:**
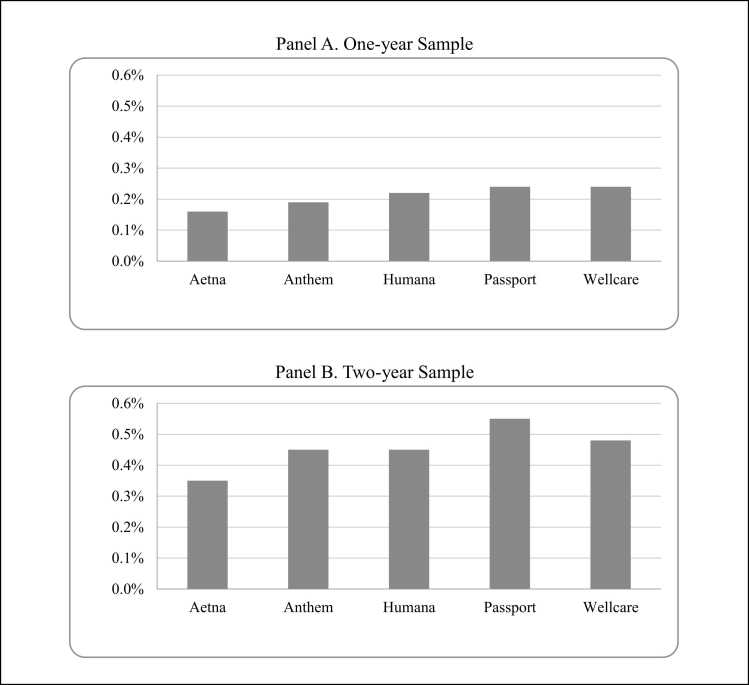
Average percentage of beneficiaries diagnosed with AUD and Receiving MAUD, by MCO plan (2017–2019). Note: Average figures for the one-year sample cover newly-enrolled individuals for the years 2017–2019 while those for the two-year sample cover newly-enrolled individuals for the years 2017–2018.

In the one-year sample, the percentage of beneficiaries who were diagnosed with AUD and received MAUD was highest for the Passport and Wellcare plans (approximately 0.24 %) (Fig. 1) and lowest for Aetna (0.16 %). For Anthem and Humana, 0.19 % and 0.22 % of beneficiaries were diagnosed with AUD and received MAUD, respectively. In the two-year sample, Passport had the highest percentage of beneficiaries who were diagnosed with AUD and received MAUD (0.55 %) while Aetna had the lowest percentage for MAUD receipt (0.35 %). Passport and Aetna, the MCO plans with the highest and lowest percentage of beneficiaries who were diagnosed with AUD and received MAUD, were also the two MCO plans with the highest and lowest comprehensiveness scores, respectively.

Table 3 presents results for the TSLS/IV models of the probability of being diagnosed with AUD and receiving MAUD. These results include state and year fixed effects and covariates. In the two-year sample, a one unit increase in plan comprehensiveness increased the probability of diagnosis and treatment with MAUD by about 0.03 (p < .05) percentage points, which is equivalent to a 6.7 % increase relative to the mean of 0.5 %. In the one-year sample, the point estimates had the same sign but the absolute value of the effect was smaller and not statistically significant. See Appendix Table 10 for the full table of results, including the F-statistics for the first stages (greater than 1000 in each case) which signal the strength of our instrument, and see Appendix Table 11 for covariate results.

**Table 3:** Results of two-stage least squares/instrumental variable models predicting the probability of AUD diagnosis and MAUD receipt.

	**One-year Sample**		
	**AUD Diagnosis and MAUD Receipt (%)**	**Difference (Percentage Points)**	**Difference (%)**
**MCO Plan Comprehensiveness**	0.2 %	0.010	5.0 %
	**Two-year Sample**		
	**AUD Diagnosis and MAUD Receipt (%)**	**Difference (Percentage Points)**	**Difference (%)**
**MCO Plan Comprehensiveness**	0.5 %	0.033⁎⁎	6.7 %

Note: The table presents estimates from a two-stage least squares/instrumental variable model. In the first stage, we regressed *realized* comprehensiveness on *assigned* comprehensiveness. In the second stage, to assess the impact of realized AUD comprehensiveness, we regressed downstream outcomes (AUD diagnosis and MAUD receipt) on predicted comprehensiveness from the first stage. All regression models controlled for sex, race and ethnicity, age, household size, and county and year fixed effects. ^⁎^*p* < 0.10, ^⁎⁎^*p* < 0.05, ^⁎⁎⁎^*p* < 0.01.

## 4 Discussion

Our results indicate that providing comprehensive coverage of AUD treatment services and medications increased the probability of a beneficiary receiving MAUD. In particular, among Medicaid beneficiaries continuously enrolled for two years, increasing MCO plan comprehensiveness of the least comprehensive plan to match the most comprehensive plan would increase the probability of receiving MAUD by as much as 47 %. Our results suggest that MCO plan comprehensiveness of coverage for AUD treatment has important implications for improving MAUD receipt – an outcome with significant implications given that AUD is an underdiagnosed and undertreated health condition in the United States. Further, the increase in the probability of MAUD receipt due to comprehensive benefits is clinically meaningful, particularly given the low rates of MAUD receipt among beneficiaries with AUD. A back of the envelope calculation indicates that 785 additional Medicaid beneficiaries (of the 981,051 beneficiaries aged 18–64 enrolled in Kentucky Medicaid in 2019) would receive MAUD in 2019 if all MCO plans were as comprehensive as the most comprehensive MCO plan.

While our results showed low rates of MAUD receipt among Kentucky Medicaid beneficiaries during the study period (0.2–0.5 %), our estimates were slightly higher than the estimate of 0.1 % reported in the 2019 National Survey on Drug Use and Health (NSDUH) (Center for Behavioral Health Statistics and Quality, 2019). This difference is consistent with general differences between Medicaid claims and NSDUH survey data, i.e., when a beneficiary is diagnosed with AUD in Medicaid, it is with the intention to treat the condition, while NSDUH survey data capture AUD among individuals in the broader population and the survey identifies AUD based on self-reported symptomology, regardless of the intention to treat the condition. Additionally, when we compare our findings for Kentucky Medicaid beneficiaries to a study of MAUD receipt among Kentucky Medicaid enrollees by Hu et al. (2022), our estimate of 0.2 % is comparable to their estimate of 0.3 %. The slightly higher estimate from the Hu et al. study is likely due to differences in study design and the study’s inclusion of topiramate (which is sometimes prescribed off-label for MAUD).

Improving the comprehensiveness of AUD treatment coverage provides beneficiaries with more options for AUD treatment, and consequently, may accelerate initiation of MAUD. Since MAUD is rarely initiated in primary care settings, receipt of MAUD often hinges on access to other AUD treatment services, such as detoxification and psychosocial therapies delivered in inpatient and outpatient treatment settings (Busch et al., 2025). For example, warm handoffs following detoxification can introduce individuals to MAUD – a relationship facilitated by close coordination among treatment providers working within an integrated system of care (Timko et al., 2016). In addition, detoxification prior to acamprosate or naltrexone has been shown to be associated with improved outcomes in terms of fewer cravings and heavy drinking (Maisel et al., 2013). Moreover, given that psychosocial therapy is one of the primary treatment modalities recommended for the treatment of AUD, psychosocial treatments can provide an opening for the introduction of MAUD (Kranzler, 2023). Beyond serving as gateway to MAUD receipt, psychosocial therapy provided in both inpatient and outpatient treatment settings, such as cognitive behavioral therapy, has been shown to improve efficacy of AUD treatment (Knox et al., 2019, Magill et al., 2019). Further, the combination of psychosocial therapy and MAUD is shown to enhance AUD treatment outcomes, particularly in terms of preventing recurrence and maintaining abstinence (Agabio et al., 2023).

Yet, our study findings revealed wide variation in plan coverage of AUD treatment services. Many MCO plans in our study did not provide any coverage for important AUD treatment services such as intensive outpatient and residential services. Given this finding, one important strategy to improve receipt of AUD treatment is to expand the comprehensiveness of AUD treatment service coverage among Medicaid MCOs. AUD is a condition that responds to both psychosocial interventions and MAUD (Abraham et al., 2020, Brewer et al., 2000, Chick et al., 1992, Fuller and Gordis, 2004, Garbutt et al., 2005, Klemperer et al., 2018, Kranzler, 2023, Kranzler et al., 2004, Lyon, 2017, Maisel et al., 2013, O’Malley et al., 2007, O’Malley et al., 1992, Oslin et al., 2015, Rösner et al., 2010a, 2010b; Soyka and Chick, 2003; Suh et al., 2006; Volpicelli et al., 1992). In fact, given that there are no blockbuster medications for the treatment of AUD (in contrast to opioid use disorder), having access to psychosocial therapy is critically important.

Our findings also highlight the importance of continuous Medicaid enrollment. In this study, we found that the percentage of beneficiaries receiving MAUD was substantially higher among beneficiaries continuously enrolled for two years, compared to beneficiaries continuously enrolled for only one year (0.5 % compared to 0.2 % receiving MAUD, respectively). Further, results showed that higher levels of MCO plan comprehensiveness were significantly associated with an increased probability of receiving MAUD for the two-year sample (i.e., beneficiaries continuously enrolled for two years), but not the one-year sample. Given high rates of churn and disenrollment in the Medicaid program due to administrative barriers to beneficiary reenrollment, a substantial percentage of Medicaid-eligible individuals lose coverage every year. In 2018, roughly 7 % of adult Medicaid beneficiaries across the United States were affected by Medicaid churn due to disenrollment and reenrollment barriers (Corallo and Garfield, 2021). The findings of this study suggest this could impact continuity of care for AUD.

### 4.1 Limitations

This study has several limitations. First, we used a straightforward measure of plan comprehensiveness by summing the number of AUD treatment services and MAUD. However, we acknowledge that there are several ways to measure MCO plan comprehensiveness, and this measure considers all treatment services and medications equally. Using this summative measure allowed us to take a first step toward understanding the relationship between MCO plan features and AUD treatment outcomes, though future research should consider whether some types of AUD treatment are more important to cover in the benefit package in terms of impacting AUD outcomes. Second, a substantial number of beneficiaries had missing values for race/ethnicity. To address this issue, we created a dichotomous variable for missing race/ethnicity, and included it in our models. Third, we limited our analyses to the four FDA-approved medications for the treatment of AUD (acamprosate, disulfiram, injectable naltrexone, oral naltrexone) and did not include medications prescribed off-label for AUD treatment such as topiramate and baclofen. Fourth, our study focused on newly enrolled beneficiaries which may limit the generalizability of our results to all Medicaid managed care beneficiaries. Fifth, our study period encompasses episodes that occurred between 2016 and 2019. While Kentucky Medicaid MCO plan coverage has changed quite modestly since the study period, we found no substantial changes in coverage of AUD treatment services or medications (e.g., in two plans, coverage remained the same, and in three plans, one additional AUD treatment modality was covered). Thus, our results likely reflect current conditions in the state. Finally, since we did not have access to administrative data from Kentucky, we used TAF enrollment data available from CMS to identify beneficiaries’ auto-assigned plan and plan of choice, for those who chose to switch plans within a 90-day period following initial enrollment. We are confident in our estimates, with rates of switching that are similar to those reported by Marton et al. (2017) study, which used administrative data (Marton et al., 2017).

## 5 Conclusion

Medicaid managed care plays a key role in shaping the delivery and quality of AUD treatment in the United States. Our results suggest that MCO plan comprehensiveness of AUD coverage has important implications for the receipt of MAUD. Policies to encourage MCO plans to offer more comprehensive coverage of AUD treatment could lead to important increases in receipt of MAUD among Medicaid beneficiaries.

## CRediT authorship contribution statement

**Miguel Antonio Garcia Estrada:** Writing – review & editing, Writing – original draft, Methodology, Formal analysis, Conceptualization. **Shelby R. Steuart:** Writing – review & editing, Methodology, Conceptualization. **Christina M. Andrews:** Writing – review & editing, Supervision, Funding acquisition, Conceptualization. **Colleen M. Grogan:** Writing – review & editing, Funding acquisition, Conceptualization. **Olivia M. Hinds:** Writing – review & editing, Conceptualization. **Emily C. Lawler:** Writing – review & editing, Methodology, Conceptualization. **Felipe Lozano-Rojas:** Writing – review & editing, Methodology, Conceptualization. **Melissa A. Westlake:** Writing – review & editing, Conceptualization. **Lauren Peterson:** Writing – review & editing, Conceptualization. **Coady Wing:** Writing – review & editing, Methodology, Formal analysis, Conceptualization. **Amanda J. Abraham:** Writing – review & editing, Supervision, Funding acquisition, Conceptualization.

## Declaration of Competing Interest

The authors declare that they have no known competing financial interests or personal relationships that could have influenced the work reported in this paper.

## Footnotes

Appendix A Supplementary data associated with this article can be found in the online version at doi:10.1016/j.dadr.2025.100374.
