# Corrigendum to “Relationship between Medicaid coverage design and receipt of medication for alcohol use disorder (MAUD): Probability of receipt increases based on comprehensiveness of plan” [Drug Alcohol Depend. Rep. 16 (2025) 100374]

**DOI:** 10.1016/j.dadr.2025.100382

**Published:** 2025-12-04

**Authors:** Miguel Antonio Garcia Estrada, Shelby R. Steuart, Christina M. Andrews, Colleen M. Grogan, Olivia M. Hinds, Emily C. Lawler, Felipe Lozano-Rojas, Melissa A. Westlake, Lauren Peterson, Coady Wing, Amanda J. Abraham

**Affiliations:** a Department of Public Administration and Policy, University of Georgia, United States; b Crown Family School of Social Work, Policy, and Practice, The University of Chicago, United States; c Department of Health Services Policy and Management, Arnold School of Public Health, University of South Carolina, United States; d Center for Health Administration Studies, The University of Chicago, United States; e National Bureau of Economic Research, United States; f School of Public and Environmental Affairs, Indiana University, United States

The authors would like to request the addition of the following funding statement:

## Funding

This research was supported by the 10.13039/100000027National Institute on Alcohol Abuse and Alcoholism of the 10.13039/100000002National Institutes of Health under Award Number R01AA029097 and the National Institute on Drug Abuse of the National Institutes of Health under Award Number R01DA052425. The funding source had no role in the design and conduct of the study; collection, management, analysis, and interpretation of the data; preparation, review, or approval of the manuscript; and decision to submit the manuscript for publication.

The authors would like to apologise for any inconvenience caused.

DOI of original article: https://doi.org/10.1016/j.dadr.2025.100374

