# Supplementary material for: Relationship between Medicaid coverage design and receipt of medication for alcohol use disorder (MAUD): Probability of receipt increases based on comprehensiveness of plan
Source: Drug Alcohol Depend Rep. 2025 Aug 22;16:100374. doi: 10.1016/j.dadr.2025.100374 (PMC12410402; doi:10.1016/j.dadr.2025.100374)
Supplement: Supplementary file 1 — Supplementary material [file mmc1.docx]

**APPENDIX**

**Appendix Table 1. Sample Size by Year**

| **Enrollment Year** | **One-year** | **Two-year** | **Attrition** |
| --- | --- | --- | --- |
| 2017 | 84,547 | 69,747 | 14,800 (17.5%) |
| 2018 | 62,016 | 49,739 | 12,277 (19.8%) |
| 2019 | 55,667 | N/A |  |
| Resulting Sample Size | 202,230 | 119,486 |  |

**Appendix Table 2. Assigned and Realized Plans: Two-year Sample**

| **First Year Assigned Plan** | **2^nd^ Year Realized Plan** | | | | | **Total** |
| --- | --- | --- | --- | --- | --- | --- |
|  | **Aetna** | **Anthem** | **Humana** | **Passport** | **Wellcare** |  |
| Aetna | 86.6% | 2.6% | 2.0% | 5.2% | 3.7% | 9,949 |
| Anthem | 1.3% | 88.8% | 2.3% | 3.2% | 4.5% | 29,016 |
| Humana | 1.1% | 2.9% | 88.2% | 3.7% | 4.1% | 22,049 |
| Passport | 1.0% | 2.5% | 1.8% | 91.0% | 3.7% | 29,688 |
| Wellcare | 0.7% | 1.3% | 1.3% | 2.4% | 94.4% | 28,785 |
| Total | 9,722 | 27,759 | 21,237 | 29,942 | 30,827 | 119,487 |

**Appendix Table 3. Cohen’s D: One-year Sample (Two Largest Urban Counties)**

|  | (1) | (2) | (3) | (4) | (5) |
| --- | --- | --- | --- | --- | --- |
|  | Aetna | Anthem | Humana | Passport | Wellcare |
| Age | 0.032 | 0.041 | 0.035 | 0.028 | 0.060 |
| Female | 0.015 | 0.020 | 0.015 | 0.043 | 0.082 |
| Household Size | 0.034 | 0.079 | 0.044 | 0.077 | 0.024 |
| Hispanic | 0.045 | 0.119 | 0.040 | 0.054 | 0.088 |
| Black | 0.046 | 0.042 | 0.040 | 0.070 | 0.054 |
| AAPI | 0.025 | 0.032 | 0.011 | 0.019 | 0.005 |
| Native American | 0.007 | 0.011 | 0.001 | 0.005 | 0.002 |
| White | 0.033 | 0.098 | 0.002 | 0.029 | 0.051 |
| Unknown Race | 0.110 | 0.004 | 0.057 | 0.077 | 0.051 |
| Primary Language Spanish | 0.047 | 0.137 | 0.053 | 0.070 | 0.073 |
| Primary Language English | 0.046 | 0.138 | 0.054 | 0.070 | 0.072 |
| 0-100% FPL | 0.016 | 0.021 | 0.012 | 0.016 | 0.014 |
| 101-133% FPL | 0.012 | 0.015 | 0.018 | 0.016 | 0.013 |
| 134-150% FPL | 0.005 | 0.012 | 0.003 | 0.006 | 0.006 |
| 151-200% FPL | 0.027 | 0.015 | 0.001 | 0.008 | 0.024 |
| 201-255% FPL | 0.002 | 0.005 | 0.016 | 0.008 | 0.004 |
| 256-300% FPL | 0.009 | 0.019 | 0.016 | 0.002 | 0.036 |
| 301-400% FPL | 0.058 | 0.001 | 0.001 | 0.012 | 0.024 |
| over 401% FPL | 0.012 | 0.012 | 0.012 | 0.007 | 0.012 |
| Veterans | 0.021 | 0.036 | 0.003 | 0.003 | 0.064 |
| US Citizens | 0.086 | 0.171 | 0.070 | 0.142 | 0.046 |
| Observations | 4,749 | 10,709 | 8,962 | 19,031 | 6,641 |

**Appendix Table 4. Cohen’s D: One-year Sample (All Other Counties)**

|  | (1) | (2) | (3) | (4) | (5) |
| --- | --- | --- | --- | --- | --- |
|  | Aetna | Anthem | Humana | Passport | Wellcare |
| Age | 0.002 | 0.030 | 0.052 | 0.016 | 0.054 |
| Female | 0.041 | 0.042 | 0.016 | 0.009 | 0.044 |
| Household Size | 0.101 | 0.035 | 0.051 | 0.016 | 0.046 |
| Hispanic | 0.026 | 0.021 | 0.029 | 0.015 | 0.018 |
| Black | 0.003 | 0.013 | 0.008 | 0.003 | 0.021 |
| AAPI | 0.015 | 0.016 | 0.009 | 0.013 | 0.033 |
| Native American | 0.003 | 0.005 | 0.004 | 0.003 | 0.006 |
| White | 0.033 | 0.002 | 0.052 | 0.038 | 0.059 |
| Unknown Race | 0.041 | 0.021 | 0.076 | 0.043 | 0.100 |
| Primary Language Spanish | 0.012 | 0.016 | 0.040 | 0.018 | 0.027 |
| Primary Language English | 0.011 | 0.016 | 0.039 | 0.018 | 0.027 |
| 0-100% FPL | 0.005 | 0.030 | 0.006 | 0.001 | 0.026 |
| 101-133% FPL | 0.010 | 0.023 | 0.005 | 0.002 | 0.021 |
| 134-150% FPL | 0.009 | 0.012 | 0.008 | 0.001 | 0.003 |
| 151-200% FPL | 0.006 | 0.002 | 0.008 | 0.008 | 0.012 |
| 201-255% FPL | 0.010 | 0.011 | 0.011 | 0.007 | 0.001 |
| 256-300% FPL | 0.020 | 0.014 | 0.002 | 0.004 | 0.015 |
| 301-400% FPL | 0.006 | 0.007 | 0.009 | 0.005 | 0.007 |
| over 401% FPL | 0.008 | 0.008 | 0.001 | 0.015 | 0.015 |
| Veterans | 0.005 | 0.012 | 0.016 | 0.001 | 0.021 |
| US Citizens | 0.051 | 0.035 | 0.016 | 0.011 | 0.043 |
| Observations | 13,082 | 38,470 | 28,655 | 31,078 | 40,853 |

**Appendix Table 5. Cohen’s D: Two-year Sample (Two Largest Urban Counties)**

|  | (1) | (2) | (3) | (4) | (5) |
| --- | --- | --- | --- | --- | --- |
|  | Aetna | Anthem | Humana | Passport | Wellcare |
| Age | 0.015 | 0.015 | 0.016 | 0.002 | 0.055 |
| Female | 0.014 | 0.033 | 0.022 | 0.038 | 0.036 |
| Household Size | 0.027 | 0.077 | 0.057 | 0.084 | 0.031 |
| Hispanic | 0.015 | 0.088 | 0.016 | 0.050 | 0.014 |
| Black | 0.070 | 0.066 | 0.048 | 0.089 | 0.044 |
| AAPI | 0.020 | 0.037 | 0.009 | 0.032 | 0.012 |
| Native American | 0.021 | 0.017 | 0.003 | 0.008 | 0.000 |
| White | 0.054 | 0.086 | 0.029 | 0.016 | 0.017 |
| Unknown Race | 0.137 | 0.021 | 0.089 | 0.114 | 0.056 |
| Primary Language Spanish | 0.019 | 0.113 | 0.024 | 0.068 | 0.005 |
| Primary Language English | 0.017 | 0.114 | 0.023 | 0.068 | 0.006 |
| 0-100% FPL | 0.006 | 0.015 | 0.012 | 0.008 | 0.015 |
| 101-133% FPL | 0.019 | 0.014 | 0.024 | 0.010 | 0.014 |
| 134-150% FPL | 0.005 | 0.003 | 0.002 | 0.002 | 0.010 |
| 151-200% FPL | 0.034 | 0.017 | 0.026 | 0.001 | 0.026 |
| 201-255% FPL | 0.015 | 0.003 | 0.013 | 0.001 | 0.006 |
| 256-300% FPL | 0.057 | 0.010 | 0.005 | 0.002 | 0.029 |
| 301-400% FPL | 0.051 | 0.017 | 0.000 | 0.014 | 0.008 |
| over 401% FPL | 0.015 | 0.015 | 0.015 | 0.009 | 0.015 |
| Veterans | 0.011 | 0.027 | 0.004 | 0.012 | 0.088 |
| US Citizens | 0.079 | 0.144 | 0.065 | 0.126 | 0.045 |
| Observations | 4,749 | 10,709 | 8,962 | 19,031 | 6,641 |

**Appendix Table 6. Cohen’s D: Two-year Sample (All Other Counties)**

|  | (1) | (2) | (3) | (4) | (5) |
| --- | --- | --- | --- | --- | --- |
|  | Aetna | Anthem | Humana | Passport | Wellcare |
| Age | 0.011 | 0.028 | 0.041 | 0.024 | 0.038 |
| Female | 0.018 | 0.044 | 0.016 | 0.009 | 0.051 |
| Household Size | 0.124 | 0.036 | 0.060 | 0.009 | 0.040 |
| Hispanic | 0.026 | 0.021 | 0.008 | 0.009 | 0.008 |
| Black | 0.013 | 0.012 | 0.011 | 0.006 | 0.017 |
| AAPI | 0.005 | 0.016 | 0.012 | 0.020 | 0.034 |
| Native American | 0.008 | 0.001 | 0.004 | 0.008 | 0.003 |
| White | 0.051 | 0.005 | 0.066 | 0.038 | 0.065 |
| Unknown Race | 0.058 | 0.024 | 0.087 | 0.042 | 0.103 |
| Primary Language Spanish | 0.006 | 0.023 | 0.022 | 0.027 | 0.009 |
| Primary Language English | 0.007 | 0.023 | 0.021 | 0.027 | 0.008 |
| 0-100% FPL | 0.009 | 0.025 | 0.011 | 0.007 | 0.024 |
| 101-133% FPL | 0.013 | 0.018 | 0.003 | 0.002 | 0.019 |
| 134-150% FPL | 0.000 | 0.011 | 0.020 | 0.008 | 0.003 |
| 151-200% FPL | 0.009 | 0.008 | 0.001 | 0.008 | 0.011 |
| 201-255% FPL | 0.008 | 0.010 | 0.008 | 0.004 | 0.004 |
| 256-300% FPL | 0.006 | 0.009 | 0.004 | 0.004 | 0.008 |
| 301-400% FPL | 0.012 | 0.003 | 0.009 | 0.011 | 0.003 |
| over 401% FPL | 0.011 | 0.011 | 0.001 | 0.019 | 0.019 |
| Veterans | 0.010 | 0.010 | 0.015 | 0.000 | 0.022 |
| US Citizens | 0.027 | 0.042 | 0.016 | 0.017 | 0.036 |
| Observations | 13,082 | 38,470 | 28,655 | 31,078 | 40,853 |

**Appendix Table 7. Models Predicting the Effect of MCO Plan Comprehensiveness on AUD Diagnosis and MAUD Receipt (Subpopulation Analysis using the TSLS/IV Model: Two Largest Urban Counties, All Other Counties)**

| **Outcome Variable** | **One-year Sample** | | |
| --- | --- | --- | --- |
|  | **Main specification** | **Two largest urban counties** | **All other counties** |
| Realized Plan Comprehensiveness | 0.0000904 | 0.000199 | 0.0000454 |
|  | (0.0000701) | (0.000127) | (0.0000562) |
| County and Year FEs | X | X | X |
| *First Stage Instrument* |  |  |  |
| Assigned Plan Comprehensiveness | 0.935*** | 0.947*** | 0.930*** |
|  | (0.00352) | (0.000213) | (0.00281) |
| F-statistic for IV in first stage | 70627.4 | 19818673 | 109341.1 |
| N | 202,230 | 50,092 | 152,138 |
| Mean of Outcome Variable | 0.00218 | 0.00224 | 0.00214 |
| SD of Outcome | 0.0464 | 0.0472 | 0.0462 |
| **Outcome Variable** | **Two-year Sample** | | |
|  | **Main specification** | **Two largest urban counties** | **All other counties** |
| Realized Plan Comprehensiveness | 0.000311** | 0.000379*** | 0.000278 |
|  | (0.000141) | (0.0000880) | (0.000197) |
| County and Year FEs | X | X | X |
| *First Stage Instrument* |  |  |  |
| Assigned Plan Comprehensiveness | 0.707*** | 0.784*** | 0.677*** |
|  | (0.0188) | (0.000692) | (0.00773) |
| F-statistic for IV in first stage | 1416.198 | 1282160 | 7657.145 |
| N | 119,486 | 28,818 | 90,668 |
| Mean of Outcome Variable | 0.005 | 0.00527 | 0.00491 |
| SD of Outcome | 0.0679 | 0.0724 | 0.0699 |

Note: Standard errors in parentheses; SEs clustered at the county level. ^*^*p* < 0.10, ^**^ *p* < 0.05, ^***^ *p* < 0.01.

**Appendix Table 8. Models Predicting the Effect of MCO Plan Comprehensiveness on AUD Diagnosis (Intent-to-Treat/Ordinary Least Squares Model; Two-Stage Least Squares/Instrumental Variable Model)**

| Outcome Variable | One-year Sample | | | | | |
| --- | --- | --- | --- | --- | --- | --- |
|  | Uncontrolled | | County and Year FEs | | FEs and Covariates | |
|  | **ITT (1)** | **TSLS (2)** | **ITT (3)** | **TSLS (4)** | **ITT (5)** | **TSLS (6)** |
|  | **AUD Dx** | **AUD Dx** | **AUD Dx** | **AUD Dx** | **AUD Dx** | **AUD Dx** |
| Assigned Plan Comprehensiveness | 0.000145  (0.000179) |  | 0.000504  (0.000452) |  | 0.000602  (0.000533) |  |
|  |  |  |  |  |  |  |
| Realized Plan Comprehensiveness |  | 0.000153  (0.000189) |  | 0.000539  (0.000480) |  | 0.000644  (0.000567) |
|  |  |  |  |  |  |  |
| County and Year FEs |  |  | X | X | X | X |
| Covariates |  |  |  |  | X | X |
| *First Stage Instrument* |  |  |  |  |  |  |
| Assigned Plan Comprehensiveness |  | 0.949***  (0.000681) |  | 0.935***  (0.00352) |  | 0.934***  (0.00353) |
| F-statistic for IV in first stage |  | 1941276 |  | 70627.4 |  | 69925.72 |
| N | 202,230 | 202,230 | 202,230 | 202,230 | 199,841 | 199,841 |
| Mean of Outcome Variable | 0.0269 | 0.0269 | 0.0269 | 0.0269 | 0.0268 | 0.0268 |
| SD of Outcome Variable | 0.1617 | 0.1617 | 0.1617 | 0.1617 | 0.1617 | 0.1617 |
| Outcome Variable | Two-year Sample | | | | | |
|  | Uncontrolled | | County and Year FEs | | FEs and Covariates | |
|  | **ITT (1)** | **TSLS (2)** | **ITT (3)** | **TSLS (4)** | **ITT (5)** | **TSLS (6)** |
|  | **AUD Dx** | **AUD Dx** | **AUD Dx** | **AUD Dx** | **AUD Dx** | **AUD Dx** |
| Assigned Plan Comprehensiveness | 0.000119  (0.000289) |  | 0.000447  (0.000555) |  | 0.000522  (0.000618) |  |
|  |  |  |  |  |  |  |
| Realized Plan Comprehensiveness |  | 0.000190  (0.000462) |  | 0.000632  (0.000770) |  | 0.000738  (0.00856) |
|  |  |  |  |  |  |  |
| County and Year FEs |  |  | X | X | X | X |
| Covariates |  |  |  |  | X | X |
| *First Stage Instrument* |  |  |  |  |  |  |
| Assigned Plan Comprehensiveness |  | 0.625***  (0.00194) |  | 0.707***  (0.0188) |  | 0.707***  (0.0177) |
| F-statistic for IV in first stage |  | 104217 |  | 1416.198 |  | 1587.69 |
| N | 119,486 | 119,486 | 119,486 | 119,486 | 119,105 | 119,105 |
| Mean of Outcome Variable | 0.0425 | 0.0425 | 0.0425 | 0.0425 | 0.0424 | 0.0424 |
| SD of Outcome Variable | 0.2720 | 0.2720 | 0.2720 | 0.2720 | 0.2720 | 0.2720 |

Note: Standard errors in parentheses; SEs clustered at the county level. ^*^*p* < 0.10, ^**^ *p* < 0.05, ^***^ *p* < 0.01.

**Appendix Table 9. Models Predicting the Effect of MCO Plan Comprehensiveness on AUD Diagnosis and MAUD Receipt (Intent-to-Treat/Ordinary Least Squares Model)**

| **Outcome Variable** | **One-year Sample** | | |
| --- | --- | --- | --- |
|  | **No control variables** | **County and Year FEs** | **FEs and Covariates** |
|  | **(1)** | **(2)** | **(3)** |
| Assigned Plan Comprehensiveness | -0.0000303  (0.0000514) | 0.0000845  (0.0000659) | 0.0000926  (0.0000714) |
|  |  |  |  |
| County and Year FEs |  | X | X |
| Covariates |  |  | X |
| N | 202,230 | 202,230 | 199,841 |
| **Outcome Variable** | **Two-year Sample** | | |
|  | **No control variables** | **County and Year FEs** | **FEs and Covariates** |
|  | **(1)** | **(2)** | **(3)** |
| Assigned Plan Comprehensiveness | -0.0000174  (0.000101) | 0.000220**  (0.000101) | 0.000236**  (0.000104) |
|  |  |  |  |
| County and Year FEs |  | X | X |
| Covariates |  |  | X |
| N | 119,486 | 119,486 | 119,105 |

Notes: Standard errors in parentheses. * p < 0.10, ** p < 0.05, *** p < 0.01. Covariates: % Black, % Hispanic, % Missing Race, % Female, % Age 18-33, % Age 34-49, % Age 50-64, Household Size.

**Appendix Table 10. Models Predicting the Effect of MCO Plan Comprehensiveness on AUD Diagnosis and MAUD Receipt (Two-Stage Least Squares/Instrumental Variable Model)**

| **Outcome Variable** | **One-year Sample** | | |
| --- | --- | --- | --- |
|  | **No control variables** | **County and Year FEs** | **FEs and Covariates** |
|  | **(1)** | **(2)** | **(3)** |
| Realized Plan Comprehensiveness | -0.0000319  (0.0000542) | 0.0000904  (0.0000701) | 0.0000991  (0.0000759) |
|  |  |  |  |
| County and Year FEs |  | X | X |
| Covariates |  |  | X |
| *First Stage Instrument* |  |  |  |
| Assigned Plan Comprehensiveness | 0.949***  (0.000681) | 0.935***  (0.00352) | 0.934***  (0.00353) |
| F-statistic for IV in first stage | 1941276 | 70627.4 | 69925.72 |
| N | 202,230 | 202,230 | 199,841 |
| Mean of Outcome Variable | 0.00216 | 0.00216 | 0.00218 |
| SD of Outcome Variable | 0.0464 | 0.0464 | 0.0464 |
| **Outcome Variable** | **Two-year Sample** | | |
|  | **No control variables** | **County and Year FEs** | **FEs and Covariates** |
|  | **(1)** | **(2)** | **(3)** |
| Realized Plan Comprehensiveness | -0.0000278  (0.000162) | 0.000311**  (0.000141) | 0.000333**  (0.000143) |
|  |  |  |  |
| County and Year FEs |  | X | X |
| Covariates |  |  | X |
| *First Stage Instrument* |  |  |  |
| Assigned Plan Comprehensiveness | 0.625***  (0.00194) | 0.707***  (0.0188) | 0.707***  (0.0177) |
| F-statistic for IV in first stage | 104217 | 1416.198 | 1587.69 |
| N | 119,486 | 119,486 | 119,105 |
| Mean of Outcome Variable | 0.005 | 0.005 | 0.005 |
| SD of Outcome Variable | 0.0679 | 0.0679 | 0.0679 |

Notes: Standard errors in parentheses. * p < 0.10, ** p < 0.05, *** p < 0.01. The two-year sample has a smaller first-stage coefficient not because people are switching plans at the beginning of the second year but because plans on average are less generous the second year. (See Appendix Table 2 for 2nd year Assigned and Realized stickiness rates). Covariates: % Black, % Hispanic, % Missing Race, % Female, % Age 18-33, % Age 34-49, % Age 50-64, Household Size.

**Appendix Table 11. Models Predicting the Effect of MCO Plan Comprehensiveness on AUD Diagnosis and MAUD Receipt (Coefficients for Covariates in the TSLS/IV Model)**

| **Outcome Variable** | **One-year Sample** | **Two-year Sample** |
| --- | --- | --- |
| Realized Plan Comprehensiveness | 0.0000991 | 0.000333** |
|  | (0.0000759) | (0.000143) |
| *Covariates* |  |  |
| Female (Ref: Male) | -0.00196*** | -0.00367*** |
|  | (0.000208) | (0.000477) |
| Household Size | -0.000797*** | -0.00182*** |
|  | (0.0000838) | (0.000169) |
| Hispanic/Latinx (Ref: White) | -0.00200*** | -0.00432*** |
|  | (0.000287) | (0.000942) |
| Black (Ref: White) | -0.00200*** | -0.00469*** |
|  | (0.000369) | (0.000812) |
| Race Unknown/Missing (Ref: White) | -0.00160*** | -0.00342*** |
|  | (0.000274) | (0.000590) |
| Age 18-33 (Ref: 50-64) | -0.000294 | -0.000309 |
|  | (0.000249) | (0.000502) |
| Age 34-49 (Ref: 50-64) | 0.00177*** | 0.00424*** |
|  | (0.000359) | (0.000585) |
| County and Year FEs | X | X |
| Covariates | X | X |
| *First Stage Instrument* |  |  |
| Assigned Plan Comprehensiveness | 0.934*** | 0.707*** |
| F-statistic for IV in first stage | 69925.72 | 1587.69 |
| N | 199,841 | 119,105 |
| Mean of Outcome Variable | 0.00218 | 0.0050 |
| SD of Outcome | 0.0464 | 0.2720 |

Note: Results for covariates are for the TSLS/IV regression models with county and year fixed effects. Standard errors in parentheses and clustered at the county level. ^*^*p* < 0.10, ^**^ *p* < 0.05, ^***^ *p* < 0.01

**Appendix Table 12. Models Predicting the Effect of MCO Plan Comprehensiveness on AUD Diagnosis and MAUD Receipt (Two-Stage Residual Inclusion Model)**

| **Outcome Variable** | **One-year Sample** | |
| --- | --- | --- |
|  | **Logit Reduced Form**  **(1)** | **TSRI**  **(2)** |
| Assigned Plan Comprehensiveness (OR) | 0.986 |  |
|  | (0.0236) |  |
| Realized Plan Comprehensiveness (OR) |  | 0.986 |
|  |  | (0.0236) |
| *First Stage Instrument* |  |  |
| Assigned Plan Comprehensiveness | 2.583***  (0.002) | 2.583***  (0.002) |
| F-statistic for IV in first stage |  | 1941276 |
| N | 202,230 | 202,230 |
| Mean of Outcome Variable | 0.0022 | 0.0022 |
| SD of Outcome | 0.0464 | 0.0464 |
| **Outcome Variable** | **Two-year Sample** | |
|  | **Logit Reduced Form**  **(1)** | **TSRI**  **(2)** |
| Assigned Plan Comprehensiveness (OR) | 0.997 |  |
|  | (0.020) |  |
| Realized Plan Comprehensiveness (OR) |  | 0.995 |
|  |  | (0.020) |
| *First Stage Instrument* |  |  |
| Assigned Plan Comprehensiveness | 1.868***  (0.004) | 1.868***  (0.004) |
| F-statistic for IV in first stage |  | 104217 |
| N | 119,486 | 119,486 |
| Mean of Outcome Variable | 0.005 | 0.005 |
| SD of Outcome Variable | 0.0679 | 0.0679 |

Notes: Standard errors in parentheses. ^*^ *p* < 0.10, ^**^ *p* < 0.05, ^***^ *p* < 0.01. Estimates are based on the model specification with no control variables and fixed effects. The two-year sample has a smaller first-stage coefficient not because people are switching plans at the beginning of the second year but because plans on average are less generous the second year. (See Appendix Table 2 for 2nd year Assigned and Realized stickiness rates). Covariates: % Black, % Hispanic/Latinx, % Missing/Other Race, % Female, % Age 18-33, % Age 34-49, % Age 50-64, Household Size.
